# A Pilot Study on Qualitative Metabolomics to Characterize Lewis Lung Carcinoma in Mice

**DOI:** 10.3390/life15020202

**Published:** 2025-01-29

**Authors:** Agnieszka Stawarska, Magdalena Bamburowicz-Klimkowska, Dariusz Maciej Pisklak, Maciej Gawlak, Ireneusz P. Grudzinski

**Affiliations:** 1 Department of Toxicology and Food Science, Faculty of Pharmacy, Medical University of Warsaw, Banacha 1, 02-097 Warsaw, Poland; ireneusz.grudzinski@wum.edu.pl; 2 Department of Organic and Physical Chemistry, Faculty of Pharmacy, Medical University of Warsaw, Banacha 1, 02-097 Warsaw, Poland; dpisklak@wum.edu.pl; 3 Department of Pharmacotherapy and Pharmaceutical Care, Faculty of Pharmacy, Medical University of Warsaw, Banacha 1, 02-097 Warsaw, Poland; maciej.gawlak@wum.edu.pl

**Keywords:** metabolomic profiling, nuclear magnetic resonance, Lewis lung carcinoma, mice

## Abstract

Metabolomics is a powerful tool that can be used to identify different stages in cancer development. In this study, the metabolomic profile of Lewis lung carcinoma (LLC) was characterized in C57BL/6 mice bearing LLC tumors. Magnetic resonance spectroscopy (nuclear magnetic resonance—NMR) was applied using a 400 MHz ^1^H NMR spectrometer. Two types of metabolites (polar and non-polar) were identified on LLC based on the analysis of methanol/water and chloroform extracts collected from lung cancer samples in mice. The investigated metabolomics show that the neoplastic processes of growing LLC on mice may affect carbohydrate; alanine and glutamate; leucine and isoleucine; lysine; creatine; and choline metabolism, whereas hypoxia states were identified due to elevated lactate in lung cancer tissues. The metabolomic profile of Lewis lung carcinoma could be considered to be a valuable biomarker in translational lung cancer research.

## 1. Introduction

Malignant neoplasms are the second leading cause of mortality in the Western World and have become a major public health problem worldwide [1]. During the last few decades lung cancer has become the leader in cancer-related mortality [2], accounting for 25.3% of all cancer-related deaths in both men and women [3,4]. There are two histological subtypes of lung cancer, non-small-cell (NSCLC) and small-cell lung cancer (SCLC) [2,5], which, according to American Cancer Society, account for about 85% of total lung cancers, with adenocarcinoma being the most common subtype, and 15% of all lung cancers, respectively [6,7]. Both types are rapidly growing malignancies with a metastatic spread feature and a poor prognosis [8,9] due to frequent presentation with either metastasis or a locally advanced stage at the time of diagnosis [2]. The treatment options for lung cancer are surgery, radiation, chemotherapy, targeted therapy, and immunotherapy [10,11]. In most cases, however, lung cancer is typically treated using a combination of different strategies depending on the type, stage, and genetic characteristics of the cancer, as well as the patient’s overall health [12]. According to the National Cancer Institute at the National Institutes of Health (NIH), all patients diagnosed with NSCLC should be tested for biomarkers to determine the best treatment options.

Metabolomics involves identifying and quantifying a wide range of metabolites. Qualitative studies in metabolomics are fundamental for discovering new biomarkers, understanding complex biological systems, and identifying key metabolic changes that are important for health and diseases such as cancers. They provide essential insights into the mechanisms of disease, guide the development of biomarkers, and offer a pathway to personalized medicine. Over the last decade, there has been growing interest in the metabolomic profiling of lung cancers [13,14,15,16,17], the potential of which lies in the acquirement of an inimitable cell fingerprint in the form of the unique composition of chemical compounds characteristic of the specific kind of cell [18]. Applying the results from metabolomics analysis may increase the probability of successful tumor treatment, among others, as a consequence of the precise diagnosis and subsequent treatment. The most promising tools in metabolomic analysis, in turn, are mass spectrometry [18,19,20,21] and nuclear magnetic resonance (NMR) spectroscopy [18,21,22]. Note that ^1^H NMR is one of the basic techniques in metabolomic analysis, allowing the identification of numerous endogenous as well as exogenous metabolites in different body fluids, such as urine, plasma, or saliva [18,21,23,24,25]. Therefore, it seems reasonable to note that the ^1^H NMR technique can also be applied to cancer tissue extracts, elucidating different metabolites as valuable biomarkers in animal lung cancer studies.

Animal studies on lung cancer are frequently preformed on C57BL/6 mice transplanted with Lewis lung carcinoma (LLC), which is considered to be an accurately reproducible syngeneic mouse model for lung cancer [23,26,27]. Lewis Lung carcinoma, which originated spontaneously as a carcinoma of the lung of a C57BL mouse, was established by Dr. Margaret Lewis in 1951 and became one of the first transplantable tumors in murine models [27,28]. Based on its morphological and immunohistochemical features, LLC is classified as non-small-cell carcinoma [5]. Several studies have investigated the metabolomic profiles of Lewis lung carcinoma (LLC) and other lung cancers to understand how metabolic alterations contribute to cancer progression [21,29,30,31]. A study by Lu et al. (2015) used ^1^H NMR spectroscopy to profile the metabolic alterations in C57BL/6 mice bearing LLC tumors and treated with oxythiamine [29]. The study identified key metabolic shifts, such as an increase in lactate, alanine, and glutamine, which are indicative of enhanced glycolysis and amino acid metabolism in LLC tumors. The researchers also observed a decrease in certain lipid metabolites, suggesting alterations in lipid metabolism. This study highlighted the importance of metabolic reprogramming in LLC tumors and provided a foundation for future investigations into metabolic markers of lung cancer progression [29]. A study by Tang et al. (2019) focused on the role of lactate and amino acids in the metabolic changes in LLC tumors. Using mass spectrometry-based metabolomics, they identified increased levels of lactate and glutamate in LLC tumors compared to normal lung tissue. The authors linked elevated lactate levels to the Warburg effect, a hallmark of cancer metabolism, and suggested that glutamate metabolism might be involved in tumor cell survival and growth under hypoxic conditions [30]. The study also explored the impact of metabolic inhibitors on LLC tumor growth, further supporting the potential of targeting metabolic pathways as a therapeutic strategy [21]. In a study by Li et al. (2019), the metabolic effects of hypoxia in LLC tumors were explored. Using ^1^H NMR and isotopic labeling, the researchers observed that hypoxia-induced changes in LLC tumors led to increased lactate production, indicative of a shift toward anaerobic glycolysis. Additionally, the study identified increased levels of amino acids such as alanine and glutamine, which were proposed to help support the tumor’s metabolic needs under low-oxygen conditions. The study emphasized the role of the tumor microenvironment in driving these metabolic changes and suggested that targeting the hypoxic response could be a promising strategy for lung cancer therapy [31]. These kinds of studies collectively demonstrate that LLC and other lung cancer models exhibit significant metabolic reprogramming, with alterations in glycolysis, amino acid metabolism, lipid metabolism, and responses to hypoxia. These findings not only deepen our understanding of lung cancer biology, but also highlight potential biomarkers and therapeutic targets for clinical application. Further metabolomic profiling in preclinical models, including using techniques such as ^1^H NMR and mass spectrometry, will continue to refine our knowledge of the metabolic landscape of lung cancer and may lead to more effective strategies for diagnosis and treatment.

The Lewis lung carcinoma model, a widely used syngeneic murine model of non-small-cell lung cancer, provides a valuable system for studying tumor progression and metastasis. Despite its widespread use, limited studies have explored the full metabolomic profile of LLC tumors, particularly with respect to the complex metabolic shifts occurring during tumor growth and hypoxic stress. This study aims to fill this gap by providing an in-depth analysis of the metabolic alterations in LLC tumors, focusing on the changes in carbohydrate, amino acid, and lipid metabolism, as well as identifying markers associated with tumor hypoxia. To the best of our knowledge, this is the first study assessing the metabolomic profiling of mice bearing LLC performed using a 400 MHz ^1^H NMR system. The study shows how qualitative metabolomic data can significantly improve the experimental application of Lewis lung carcinoma and outlines the advantages and disadvantages of using ^1^H NMR in preclinical cancer studies.

## 2. Materials and Methods

Lewis lung carcinoma (LLC) cells obtained from the American Type Culture Collection (ATCC, Manassas, VA, USA) were used in this study. Frozen stock vials of the cancer cells were thawed and used throughout the study. The cells were routinely cultured at 37 °C in a humidified atmosphere with 5% CO_2_ in 50 cm^2^ flasks containing 10 mL of Dulbecco’s modified Eagle’s medium (DMEM) supplemented with 10% fetal calf serum (FCS), 2 mM L-glutamine, 100 IU/mL penicillin, and 100 µg/mL streptomycin (all from GIBCO, Paisley, UK). The medium was changed every third day. The cells were washed twice with phosphate-buffered saline (PBS) and incubated with trypsin–ethylenediamine tetra-acetic acid (EDTA) solution (0.25% trypsin, 1 mM EDTA) for 2 min at 37 °C to detach the cells. Thereafter, the complete media were then added into the flask at room temperature to inhibit the effect of trypsin. The cells were washed twice by centrifugation and re-suspended in the complete fresh media for reseeding and growing in new cultures. The cells were counted using a cell counter (Nano EnTek, Seoul, Republic of Korea) and prepared in a sterile PBS solution to be subcutaneously injected into the mice.

**Figure 1:**
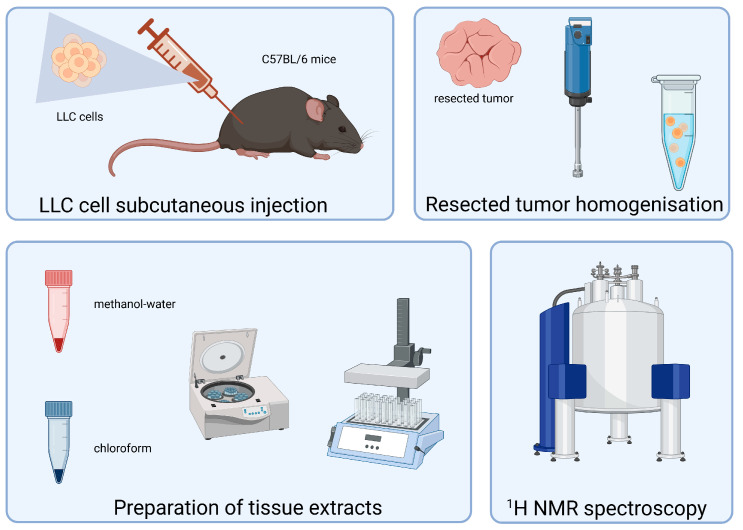
A schematic diagram illustrating the experimental workflow.

The investigations were approved by the 2nd Local Ethics Commission at the Medical University of Warsaw, which deals with experiments involving laboratory animals (No 79/2014). Six- to eight-week-old female C57BL/6JCmd (JAX^TM^) mice (n = 7) purchased from the Mossakowski Institute of Experimental and Clinical Medicine, Polish Academy of Science (Warsaw, Poland), were housed in a group of four/three animals in standard cages with free access to a standard diet and water ad libitum and were subjected to a 12 h light/dark cycle. The animals were acclimated to the animal facility for at least one week prior to the experimental procedure. For data collection, the animals in each cage were randomly numbered, and were injected subcutaneously in the right flank with 1 × 10^6^ LLC cells dispersed in 100 µL sterile PBS. The mice bearing LLC were sacrificed at three weeks post-injection, and cancer tissues were collected from the mice and further used in the ^1^H NMR metabolomic studies. In brief, the tumor samples were dissected from the mice and were homogenized in PBS through sonication, and the homogenized tissues underwent extraction through the sequential use of methanol/chloroform at a ratio of 1:3. The extraction procedure was performed with slight modifications according to the procedures previously described [32,33,34]. For data collection, the tumor samples were dissected from the mice and were homogenized in PBS at a ratio of 1:9 through sonication in a crushed ice bath. The homogenate was then divided into two parts of 1.5 mL each in centrifugation tubes. In one tube, we performed extraction with 0,5 mL of a 1:3 (*v*/*v*) ratio of ice-cold methanol/chloroform, and in the second tube, 500 mL of 1:2 (*v*/*v*) ice-cold water/methanol. The tubes were mixed for 10 min on a shaker followed by centrifugation at 13,000 rpm for 15 min at 4 °C. The bottom and top layers, respectively, were separated into other tubes, taking care not to disturb the pelleted debris. After the extraction, the samples were centrifuged, and the supernatant was evaporated under a stream of nitrogen. The remaining residues were dissolved in the deuterated solvents and placed in 5 mm NMR tubes. The polar fraction was dissolved in 0.6 mL of D_2_O and the lipid fraction in 0.6 mL of CDCl_3_, and then subjected to ^1^H NMR. A schematic diagram illustrating the experimental workflow is presented graphically in Figure 1.

The proton nuclear magnetic resonance (^1^H NMR) spectra were recorded for the methanol/water and chloroform fractions of the tumor extracts collected from the mice. The purpose of recording the high-resolution spectra was to unambiguously assign signals from the main metabolites. All spectra were recorded on a Bruker Avance DRX 400 spectrometer (Billerica, MA, USA) at a ^1^H resonance frequency of 400.13 MHz in a constant magnetic field of 9.4 T. For the D_2_O solution, an additional ^1^H selective pre-saturation pulse was applied to suppress the water signal. For CDCl_3_ solution, the standard pulse-acquisition sequence with a 90° pulse of 8 was applied. For all the spectra, 256 induction FIDs (Free Induction Decays) were collected into 8 K data points with a spectral width of 6 kHz, an acquisition time of 1.3 s, and a pulse recycle delay of 5 s. The as-obtained data were zero-filled to 16k points, and an exponential function with 0.5 Hz Lorenz line broadening was applied prior to the Fourier transform. The spectra were manually phased, and base line corrections were applied. For CDCl_3_ and D_2_O, the spectra were calibrated on a TMS signal (0 ppm) and a lactate signal (1.33 ppm), respectively.

The signal assignment of the major metabolites described below is based on literature data [34] and the Human Metabolome Database service (https://hmdb.ca), based on the signal’s chemical shift and multiplet analysis.

## 3. Results and Discussion

**Figure 2:**
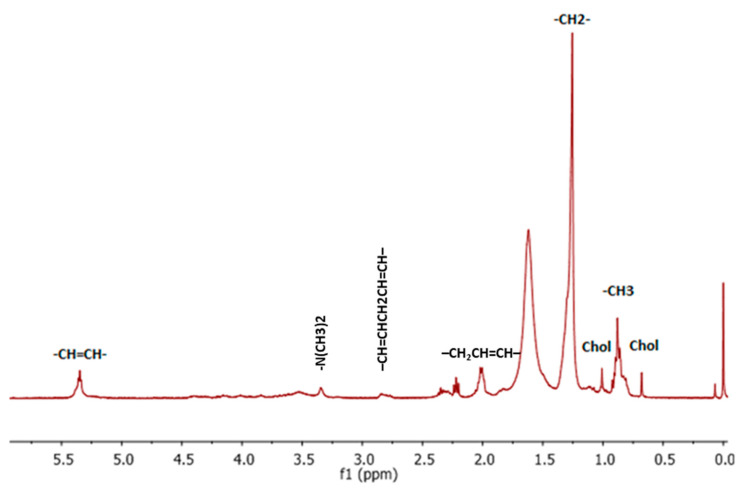
Representative ^1^H NMR spectra of chloroform extracts of LLC tumors in mice. ^1^H NMR was applied using 400 MHz NMR system.

In the present study, standard extraction techniques were applied to identify polar and non-polar metabolites in water/methanol and chloroform lung cancer tissue extracts. The signal assignment of the major metabolites was performed based on chemical shifts and multiple analyses. Some sample spectra from the analyzed extracts are provided in the Supplementary Materials (Figures S1–S7). In the ^1^H NMR spectra of the CDCl_3_ fraction, fatty acid (FA) signals were mainly identified (Figure 2). Fatty acids play a multifaceted role in cancer progression by affecting energy metabolism, cellular structure, inflammatory processes, and key signaling pathways [35,36,37,38]. The reprogramming of lipid metabolism in cancer cells allows them to thrive in the challenging tumor microenvironment [39,40]. Several types of cancer cells, including lung cancer [17,41,42], breast cancer [43], prostate cancer [44], ovarian cancer [45], colorectal cancer [39], leukemia [46], liver cancer [47], and glioblastoma [48], are known to be particularly dependent on fatty acids for their survival, growth, and metastasis. Tumor cells can increase de novo lipogenesis, fatty acid uptake, and FA oxidation for energy production and lipid accumulation [49]. While lipids are required for elevated cell proliferation, certain lipid species can affect cellular stress and must be maintained in a certain balance. As is already known, the degree of fatty acid unsaturation plays a key role in balance, and the saturation level of cell membrane lipids is a matter of life and death for tumor cells [49]. The lipids visible in NMR spectra are triglycerides and cholesterol esters, which accumulate in lipid droplets formed in response to unfavorable conditions in the tumor microenvironment [23,50]. On the one hand, high lipid levels in cancer are associated in necrosis processes [51] and apoptosis [50]. In the present study, the broad asymmetric signal at 1.25 ppm was assigned to the methylene (-CH_2_^−^) groups of the lipid acids, and overlapping narrow signals in the range of 0.76–0.93 were assigned to the methyl terminal groups (-CH_3_). The relative intensity of each signal was 3.6:1, indicating the presence of short-chain fatty acids. It was also possible to identify signals from the methylene groups adjacent to the carboxyl groups, whose position in the spectrum was about 2.2 ppm. The unsaturated fatty acids were identified based on the diagnostic resonances of allylic (–CH_2_CH=CH–) and olefinic (–CH=CH–) protons at 1.95–2.10 and 5.30–5.40 ppm, respectively. The presence of polyunsaturated fatty acids was assessed based on the presence of bis-allylic protons (–CH=CHCH_2_CH=CH–) at 2.72–2.88 ppm. According to Griffin et al., apoptosis in cancer cells may be particularly associated with an increase in the amount of unsaturated lipids that are not membrane-associated, but as-observed polyunsaturated fatty acids are most likely components of cytoplasmic lipid vesicles [52]. These kinds of lipids are probably released as part of the apoptotic process during the collapse of membrane-bound vesicles, for example, the mitochondrial membrane [51,52]. The literature also shows that fatty acid β-oxidation plays an important role in melanoma metastasis, and cancer metastases can be activated during the process of cell migration, invasion, and angiogenesis by modulating protein phosphorylation and acetylation, which are controlled by specific genes such as MMP-2 and MMP-9 [53]. Additionally, characteristic narrow signals at 0.68 ppm and 1.01 ppm of the methyl group of cholesterol were identified in the spectrum. In the lipid extracts, choline resonance arises mainly from phosphatidyl-choline molecules, and N(CH_3_)_2_ resonance can be observed at 3.30–3.40 ppm. It was found that phosphatidyl-choline is formed from choline, which, in turn, is formed through the action of glycerophosphocholine, and breaks down glycerophosphocholine into choline and -3-phosphoglycerol, whereas 3-phosphoglycerate is an important source of one-carbon units for nucleotide synthesis, via serine [52]. Furthermore, choline plays an important role in choline-mediated single-carbon metabolism and the methionine/homocysteine cycle since it takes part in the methylation reaction after oxidation to betaine [21]. Note that the choline-rich structures are the main components of the cell membrane [54,55]. Anomalous choline metabolism relates to aberrant membrane synthesis and tumor formation. The increase in choline-containing compounds reflects cell death (apoptosis or necrosis), and the alterations in choline detected in vitro by ^1^H NMR can be used as a prognostic factor in small-cell carcinoma [21]. On the other hand, decreased serum choline levels have been observed in lung cancer, which has been linked to the increased requirement of cancer cells for proliferation and the use of choline as a building block of membrane phospholipids [56]. Anomalous lipid metabolism has also been demonstrated in lung cancer. The results obtained by Guo et al. [57] and Yu et al. [58] suggest that significant changes in the metabolism of certain lipids, including sphingomyelin, phosphatidylcholine, and fatty acid derivatives, have been associated with lung cancer progression. Furthermore, changes in phospholipid distribution have been shown to decrease with cancer development, proliferation, angiogenesis, and metastasis [59]. In addition, studies using cell cultures, tissue, and animal models have shown that free fatty acids lead to the development, progression, and metastasis of lung cancer [60,61]. Phosphatidylcholine plays a crucial role in membrane structure in lung neoplasm cells as well as cell signaling [62].

**Figure 3:**
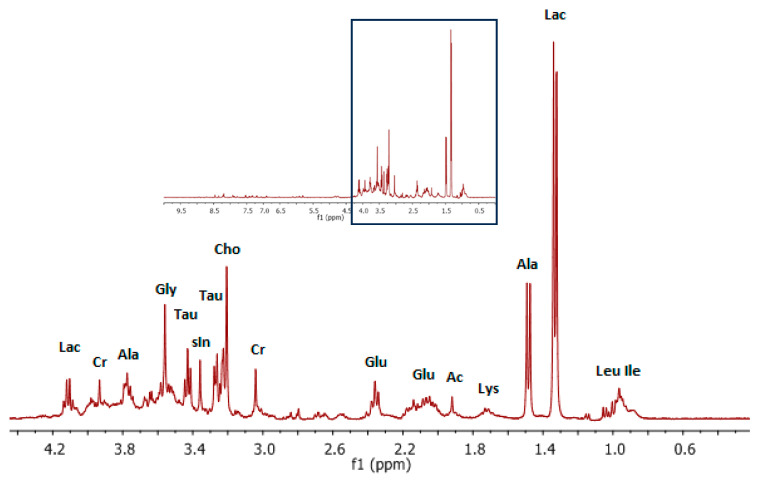
Representative ^1^H NMR spectra of water/methanol extract of LLC tumors in mice. Aliphatic region of ^1^H NMR spectra of water/methanol extract with assigned signals (Lac—lactate; Ala—alanine; Cho—choline; Cr—creatine; Glu—glutamine; glutamate; sIn—scyllo-inositol; Leu—leucine; Ile—isoleucine; Lys—lysine; Ac—acetate). ^1^H NMR was applied using 400 MHz NMR system.

In the ^1^H NMR spectrum of the water/methanol extract, the expected resonances of water-soluble cell metabolites were observed. The 400 MHz ^1^H NMR spectrum of the extract in D_2_O is presented in Figure 3. The slightly distorted signal at 4.8 ppm is a remnant of the suppressed water signal. In comparison to the chloroform extract, a large number of metabolite signals can be identified even in the aromatic region of the spectrum. The highest doublet at 1.33 ppm indicates the presence of lactate as the dominant metabolite in the sample. The presence of lactate is also confirmed by the quartet at 4.11 ppm. This clearly indicates the presence of hypoxic regions in the studied neoplasm [63]. One of the major changes found in the present study on Lewis lung cancer is the increased production of lactic acid as a result of glycolysis disorders. A large amount of lactic acid in neoplastic tissues, possibly derived from pyruvic acid, is a key factor triggering hypoxia states in solid tumors [30]. The spectra obtained on the basis of water/methanol extracts clearly indicate large amounts of lactate fractions in the tested tumor. This result clearly confirms the metabolomic profile studied in vivo, evidencing real hypoxia in cancer tissues. Note that hypoxia states are the major obstacle of chemotherapy and/or radiotherapy in solid tumors, including lung cancer [64,65,66]. The high levels of lactate, succinate, and citrate reported by many in lung tumors indicate that glycolysis and the tricarboxylic acid (TCA) cycle have been significantly altered in lung tumor tissues [24,29]. Lung cancer is often diagnosed at advanced stages, making early detection crucial for improving patient outcomes. Metabolomic profiling provides an opportunity to identify biomarkers that reflect the biochemical alterations occurring in tumors, which could be leveraged for non-invasive diagnostic techniques such as imaging or biofluid analysis. The metabolites and metabolic pathways altered in LLC tumors, such as lactate accumulation, amino acid profile changes, and lipid metabolism reprogramming, could play a key role in early detection [22]. One of the most prominent metabolic changes observed in LLC tumors is the elevation of lactate. This increase is indicative of a shift toward anaerobic metabolism, which is commonly associated with tumor hypoxia. The Warburg effect, wherein cancer cells rely on glycolysis even in the presence of oxygen, is thought to contribute to increased lactate production [30]. Elevated lactate levels can be detected in various biofluids, including blood, urine, and exhaled breath, making it a potential biomarker for early-stage lung cancer. Several studies have already highlighted the potential of lactate as a biomarker for cancer detection. For instance, elevated lactate levels have been detected in the plasma of patients with various cancers, including lung cancer. The ability to detect lactate in biofluids or via imaging could provide a valuable tool for early screening and diagnosis, enabling clinicians to intervene at earlier stages when treatment outcomes are typically more favorable [67,68,69,70].

The identified hypoxia-related changes in lung cancer could help inform strategies for overcoming hypoxia-induced therapy resistance, a common challenge in lung cancer treatment. Hypoxia, which occurs in rapidly growing tumors due to insufficient blood supply, often leads to resistance to conventional therapies such as chemotherapy and radiation [66]. By targeting the metabolic pathways associated with hypoxia, there may be potential to sensitize tumors to these therapies and improve overall therapeutic efficacy. For instance, metabolic alterations like elevated lactate levels, which are often linked to hypoxia, could serve as a target for therapeutic intervention. Utilizing metabolic inhibitors to block specific pathways, such as those involved in lactate production or anaerobic metabolism, might reduce the tumor’s ability to adapt to hypoxic conditions and enhance the effectiveness of standard treatments [65]. Additionally, targeting other hypoxia-related metabolic changes, such as altered amino acid and lipid metabolism, could further help in overcoming therapy resistance. By addressing the metabolic vulnerabilities of hypoxic tumors, it may be possible to improve the response to current therapies and achieve better clinical outcomes for patients with lung cancer [71]. According to Davidson et al., in the case of lung cancer, the contribution of glucose to the TCA cycle increases because it is converted to lactate [72]. Thus, glucose is essential for the development of this cancer. Moreover, Yang et al. reported that lung cancer cells are characterized by higher glycolysis, where lactate can be converted to pyruvate, pyruvate carboxylase activity increases, and the abundantly produced glycolytic intermediates are then incorporated into the serine metabolic pathway [73]. In this way, lactate can be used by the cells as a carbon source.

Increased metabolic rates of tumor cells require the presence of large amounts of creatine and phosphocreatine. Creatine is phosphorylated to phosphocreatine and takes part in ATP synthesis in tumor tissue. The high levels of creatine and phosphocreatine in tumors reflect their essential roles in energy storage. In breast cancer and lung metastasis models, increased levels of lactate, alanine, glutamate, and creatine are frequently observed [74]. The characteristic doublet at 1.48 ppm could be assigned to the methyl signal of alanine, as in in vivo studies, as an energy production sign in tumor cells. The important energy metabolism pathways in cancer cells require L-alanine, which, with pyruvic acid, is essential for aspartate synthesis. L-alanine is also converted from pyruvate, a large amount of which is produced by aerobic glycolysis [21]. The increase in alanine levels may result from the impaired expression of alanine transaminase, the activity of which is used to assess tissue damage and adverse events in mouse lung cancer models [74].

Deeper analysis of the aliphatic region of the spectrum (Figure 3) allows us to identify a strong singlet at 3.20 ppm which, according to the literature, could be assigned to the N(CH_3_)_2_ moiety of choline, changes in which are related with carcinogenesis and tumor progression [21]. We also found the two singlet resonances of creatine (Cr −3.03 ppm and 3.92 ppm). An intense singlet at 3.36 ppm appeared for scyllo-inositol (sIn) protons. Thus, inositol plays an important role in the regulation of cell osmotic pressure and volume and is a second messenger for intracellular signaling. Tumor levels of inositol correlate with the density of neoplastic cells, which is observed in both in vitro and in vivo NMR studies [74,75]. Furthermore, phosphorylated inositol, after conversion to diacylglycerol and inositol 1,4,5-triphosphate, can activate protein kinase C and proteolytic enzymes such as matrix metalloproteinases, which are known factors of tumor invasion [76]. Additionally, the above-reported data correspond to those obtained in humans, where Lac, GPC, PCh, Tau, Cr, and lipids turn out to be elevated in tumors [25]. The well-resolved triplet at 3.42 ppm, according to the literature, could be assigned to the taurine signal, but the second triplet at 3.25 ppm characteristic of the taurine compound is partly overlapped by choline compounds. Taurine detected in the spectrum can be associated with the abnormal proliferation of cancer cells. This was also observed in an Hmga1 mouse model of colorectal cancer [77]. On the other hand, taurine can elevate antioxidant enzyme activity, which was observed by Yu et al. on B16F10 mouse melanoma cells [78].

**Figure 4:**
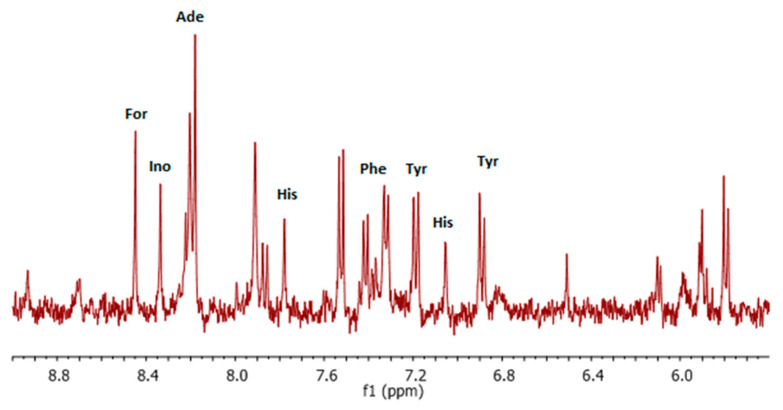
High-field region of ^1^H NMR spectrum of water/methanol extract with assigned signals. (Tyr—tyrosine; His—histidine; Phe—phenylalanine; Ade—adenine; For—formate; Ino—inosine). ^1^H NMR was applied using 400 MHz NMR system.

In the low-field region of the spectrum (Figure 4) most of signals can be assigned to aromatic amino acids. Two doublets (6.89 ppm and 7.19 ppm) can be assigned to tyrosine (Tyr), and two singlets (7.06 ppm and 7.78 ppm) confirm the presence of histidine (His). It was noted that tyrosine levels were higher in the lung cancer patient group than in the control group. The increase in the concentration of aromatic amino acids may be caused by a disorder of protein metabolism in cancer patients [21]. Otherwise, histidine, due to the fact that it releases the α-amino group, and its metabolism ends with glutamate, is one of the glucogenic amino acids [53]. Overlapped signals in the range of 7.30–7.45 ppm can be assigned to the aromatic ring of phenylalanine (Phe). Furthermore, two narrow signals at 8.44 ppm and 8.33 ppm confirm the presence of formic acid (Form) and inosine (Ino), respectively. Amino acids, especially those associated with the TCA cycle, have been suggested to be an alternative energy source for cancer cell proliferation [30]. The assignment of the remaining signals in the spectrum was ambiguous and would require the use of 2D NMR correlation spectra.

Additionally, L-glutamine is involved in the synthesis of proteins, lipids, and nucleotides, and, due to this feature, is fundamental for the proliferation and survival of cancer cells. The glutamine requirement depends on the energy requirements of the cancer cells. Increasing levels of L-glutamine are observed in lung cancer during malignant tumor progression [79]. Meanwhile, the glutamate agent, nonessential amino acid, and major excitatory neurotransmitter that is normally confined to the synaptic and presynaptic spaces of glutamatergic synapses, through conversion to glutamine with the assistance of glutamine synthetase, may participate in purine metabolism [80]. Moreover, with the participation of transaminase, glutamate can be formed from alanine or aspartate, followed by this reaction’s by-products (pyruvate and oxaloacetate) entering the TCA cycle, glycolysis, and gluconeogenesis pathways [53]. Due to the Warburg effect, pyruvic acid is converted to lactic acid in the presence of oxygen. Moreover, the rapid energy consumption of up-regulated glycolysis is consistent with the Warburg effect. An alternative method to lactic acid production is the upregulation of the glyoxal-dependent pathway and conversion of cytotoxic methylglyoxal into D-lactic acid. Furthermore, glucose can also be converted into lactic acid [81]. While taurine is a metabolite synthesized via the cysteine-sulfinic acid pathway, it is thought to have a role in cancer cells similar to inositol [82]. According to the literature, the metabolic changes mainly include increased levels of taurine, creatine, leucine, glutamate, isoleucine, aspartate, and lactate, and the degradation of arginine, inositol, and creatine, among others, as well as amino acids in neoplastic tissues [83,84]. The as-obtained metabolomic profile of Lewis lung carcinoma matched well with the literature reports for in vitro ^1^H NMR studies accurately describing the tumor burden, which is mainly characterized by hypoxia states [66,85,86,87].

The use of a 400 MHz ^1^H NMR spectrometer, while effective for identifying a broad range of metabolites, has limitations in detecting low-abundance compounds involved in fast metabolic cycles, such as ATP, ADP, NADP, NADH, and NADPH. These metabolites are critical for energy metabolism and redox regulation but were not detected in this study. Future research could benefit from utilizing higher-field-strength NMR spectrometers (e.g., 600 MHz or 800 MHz) or employing alternative techniques, such as ^31^P NMR, which would improve sensitivity for detecting nucleotides like ATP and ADP, as well as coenzymes like NAD(P)H. While we identified several key metabolites associated with the LLC tumor model, the quantification of specific metabolites, such as lactate, alanine, glutamate, and others, could be improved by employing additional analytical techniques, including mass spectrometry or more advanced NMR pulse sequences. This would allow for a more detailed understanding of the metabolic shifts in the tumor microenvironment, particularly in the context of altered energy metabolism and hypoxic conditions.

This study focused on the qualitative characterization of metabolomic profiles at a single time point. To gain a more comprehensive understanding of the metabolic changes during cancer progression, future studies should include a time-series analysis of metabolite dynamics throughout the course of LLC tumor development. Additionally, incorporating a larger sample size could help account for biological variability and enhance the statistical power of the findings. Future studies could integrate metabolomics with other OMICS approaches, such as transcriptomics and proteomics, to provide a more holistic view of the molecular mechanisms underlying LLC progression. This multi-OMICS approach could reveal key regulatory networks and identify potential therapeutic targets. Expanding this study to include human lung cancer samples, either from patient biopsies or more clinically relevant animal models, would enhance the translational potential of the findings. Comparing the metabolomic profiles of LLC in mice to those in human lung cancer would help identify conserved metabolic signatures that could serve as biomarkers for early detection and therapeutic intervention. Given the identified metabolic alterations in LLC, future research should explore how targeting specific metabolic pathways—such as the lactate dehydrogenase pathway or amino acid metabolism—could influence tumor growth and progression. This could open new avenues for therapeutic strategies aimed at disrupting the altered metabolic pathways in cancer cells. Incorporating these future directions will not only address the limitations identified in this study but also provide valuable insights for the development of targeted therapies based on tumor-specific metabolic signatures.

Lewis lung carcinoma has been used over the last seven decades as a preclinical cancer model, but it has still not been metabolically characterized. Please note that LLC was preliminarily genetically and genomically characterized in the last year by He et al. (2024) [88]. Our pilot studies give one of the first looks into the metabolomic fingerprint of LLC and should be continued.

## 4. Conclusions

These qualitative metabolomic studies were performed in C57BL/6 mice bearing Lewis lung carcinoma using the ^1^H NMR technique. Two different extraction processes, i.e., methanol/water and chloroform, were used to identify polar and non-polar metabolites in lung cancer tissues. The obtained metabolomic data showed that neoplastic changes due to LLC progression may affect carbohydrate; alanine and glutamate; leucine and isoleucine; lysine; creatine; and choline metabolism. One of the main changes found in this study is the increased glycolysis and increased production of lactic acid, which lung cancer cells produce from methylglyoxal, thus avoiding methylglyoxal-induced self-destruction. Since glucose is almost quantitatively converted to lactic acid, the tricarboxylic acid cycle works in the opposite direction, using glutamine as an alternative carbon source. In addition, alanine aminotransferase catalyzes the transfer of the amino group from glutamic acid to pyruvic acid with the release of alanine, the concentration of which increases significantly. Moreover, formed from glycine, creatine, in turn, can be phosphorylated to phosphocreatine, and is ultimately used for the synthesis of adenosine triphosphate (ATP), so it is necessary for increasing the metabolic rates of tumor cells. Myo-inositol and taurine, in increased amounts synthesized in tumors, play an important role in the regulation of cell volume and osmotic pressure. Abnormal choline metabolism has been shown to be a critical metabolic feature closely related to abnormal membrane synthesis and tumorigenesis. In most cases of malignant tumors, choline is extensively metabolized to phosphocholine. To the best of our knowledge, this is the first study assessing the metabolomic profiling of mice bearing LLC performed using a 400 MHz ^1^H NMR system. Our results are consistent with mass spectrometry-based lung cancer metabolomics studies available in the Lung Cancer Metabolome Database [89] and other recently published data obtained using LC-MS/MS techniques in humans, and confirm different groups of metabolites to be the most significant biomarkers in lung cancer [90,91,92,93]. The results also show that murine LLC tumors can be considered valuable preclinical models for human lung carcinoma.

Incorporating the observed metabolic changes into early-detection strategies could significantly impact lung cancer diagnosis and management. By utilizing non-invasive techniques that target key metabolic alterations—such as elevated lactate, altered amino acid metabolism, and changes in lipid profiles—clinicians could detect lung cancer at earlier, more treatable stages. This approach not only provides a potential pathway for early diagnosis but also opens up new avenues for developing therapeutic strategies that target the metabolic vulnerabilities of lung cancer cells. Future studies will be crucial in validating these biomarkers in clinical settings and refining techniques for their implementation in routine clinical practice.

## Data Availability

Data are available upon request from the corresponding authors.

## Supplementary Materials

The following supporting information can be downloaded at: https://www.mdpi.com/article/10.3390/life15020202/s1, Figures S1–S7: Examples of NMR spectra obtained in water/methanol lung cancer tissue extracts.
