# Supplementary material for: A Pilot Study on Qualitative Metabolomics to Characterize Lewis Lung Carcinoma in Mice
Source: Life (Basel). 2025 Jan 29;15(2):202. doi: 10.3390/life15020202 (PMC11857005; doi:10.3390/life15020202)
Supplement: Supplementary file 1 [file life-15-00202-s001.zip › life-3410010-supplementary.pdf]

Supplementary Materials

A Pilot Study on Qualitative Metabolomics to Characterize Lewis Lung Carcinoma in Mice

Agnieszka Stawarska [1,*], Magdalena Bamburowicz-Klimkowska [1,*], Dariusz Maciej Pisklak [2], Maciej Gawlak [3] and Ireneusz P. Grudzinski [1]

[1] Department of Toxicology and Food Science, Faculty of Pharmacy, Medical University of Warsaw, Banacha 1, 02-097 Warsaw, Poland; ireneusz.grudzinski@wum.edu.pl
[2] Department of Organic and Physical Chemistry, Faculty of Pharmacy, Medical University of Warsaw, Banacha 1, 02-097 Warsaw, Poland; dpisklak@wum.edu.pl
[3] Department of Pharmacotherapy and Pharmaceutical Care, Faculty of Pharmacy, Medical University of Warsaw, Banacha 1, 02-097 Warsaw, Poland; maciej.gawlak@wum.edu.pl
* Correspondence: agnieszka.stawarska@wum.edu.pl (A.S.); mjbamburowicz@wum.edu.pl (M.B.-K.); Tel.: +48-22-57-20-744 (A.S.); +48-22-57-20-760 (M.B.-K.)

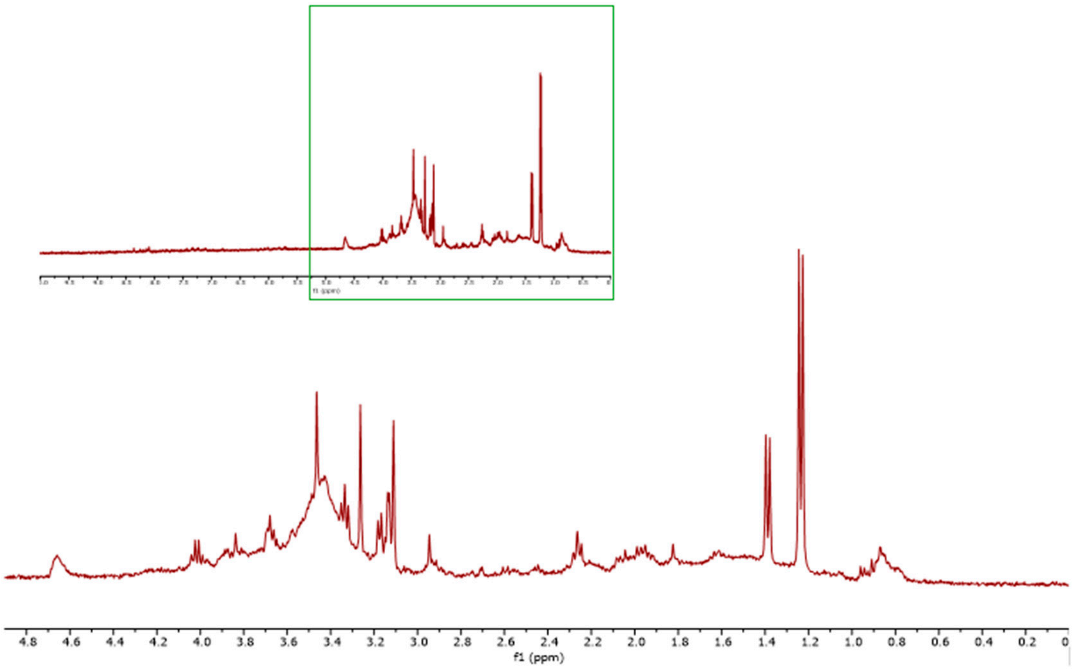

Figure S1. Representative ^{1}H NMR spectra of water/methanol extract of LLC tumor in mouse number 1.

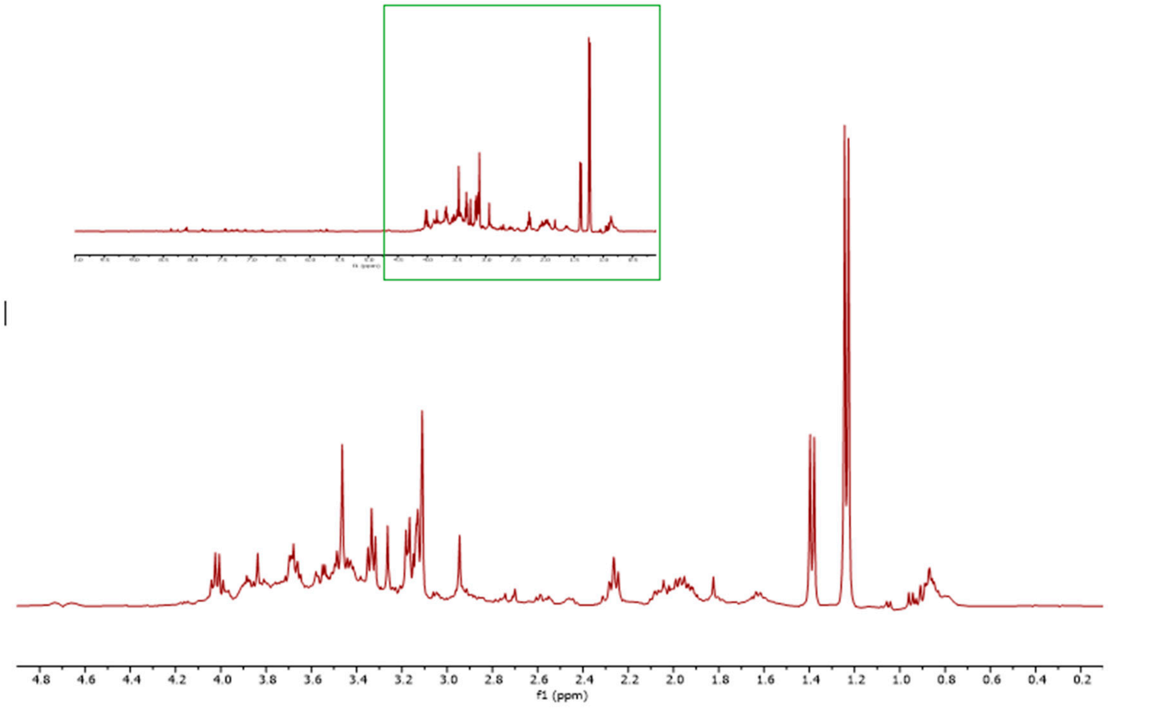

Figure S2. Representative ^{1}H NMR spectra of water/methanol extract of LLC tumor in mouse number 2.

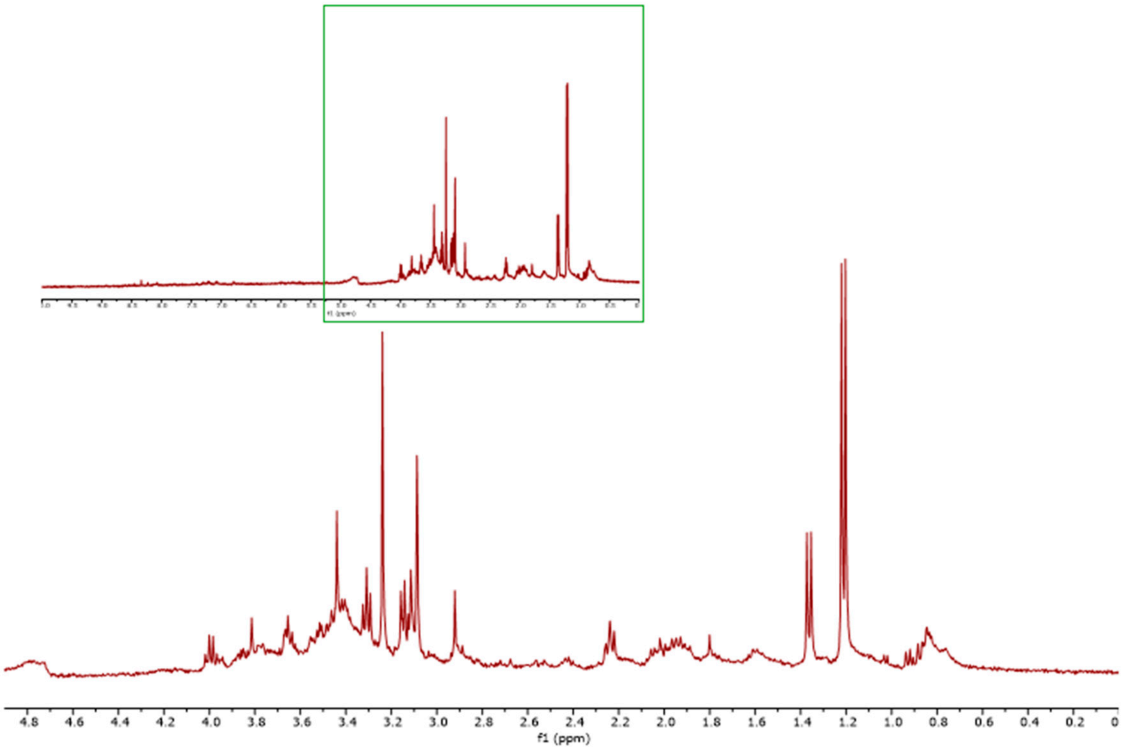

Figure S3. Representative ^{1}H NMR spectra of water/methanol extract of LLC tumor in mouse number 3.

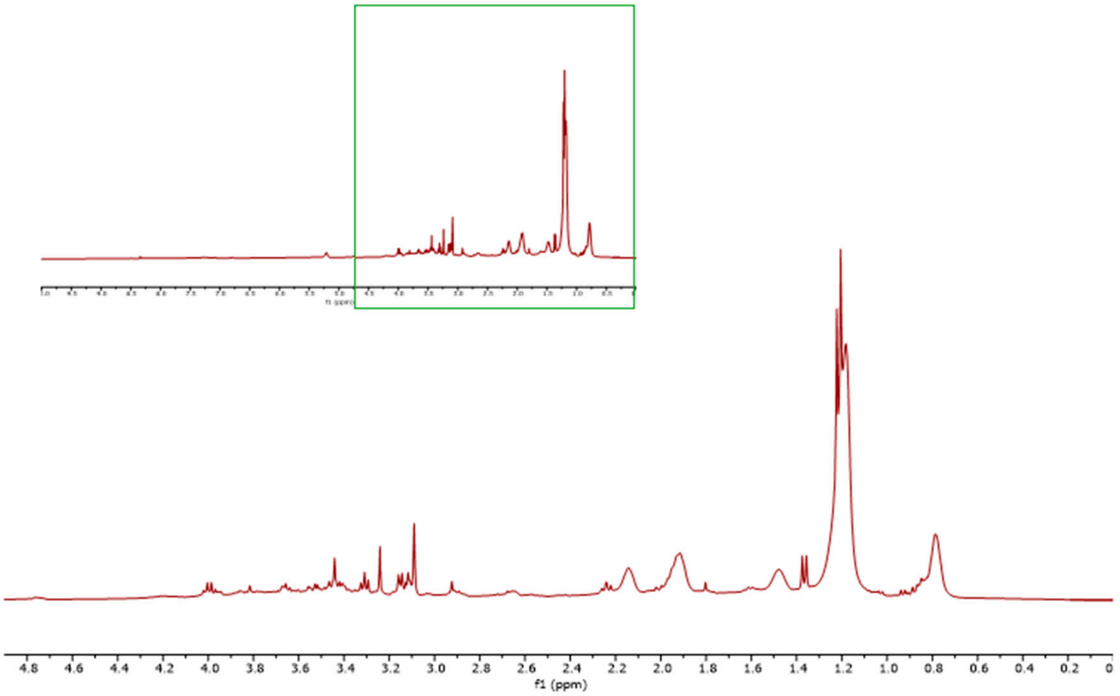

Figure S4. Representative ^{1}H NMR spectra of water/methanol extract of LLC tumor in mouse number 4.

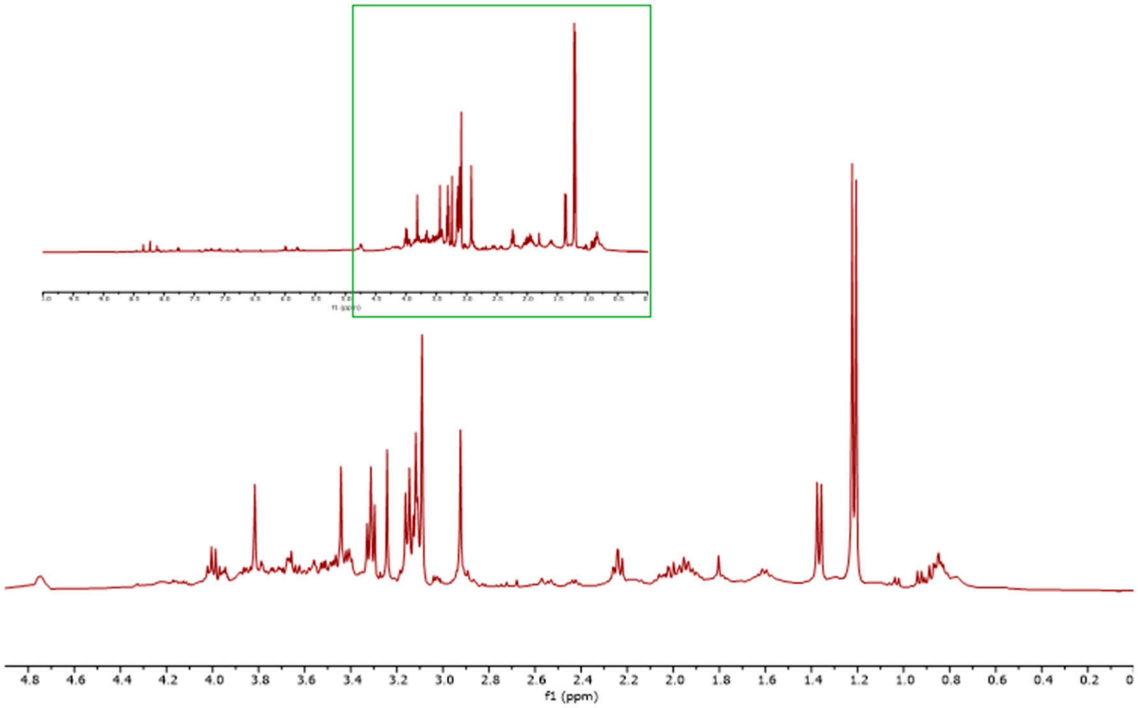

Figure S5. Representative ^{1}H NMR spectra of water/methanol extract of LLC tumor in mouse number 5.

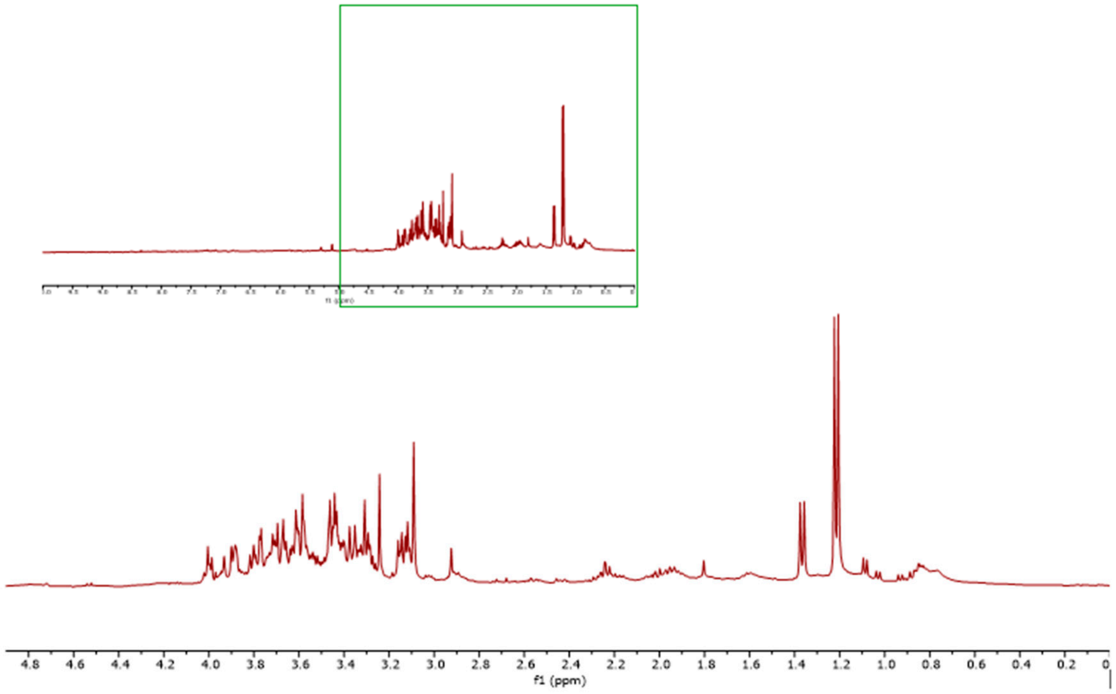

Figure S6. Representative ^{1}H NMR spectra of water/methanol extract of LLC tumor in mouse number 6.

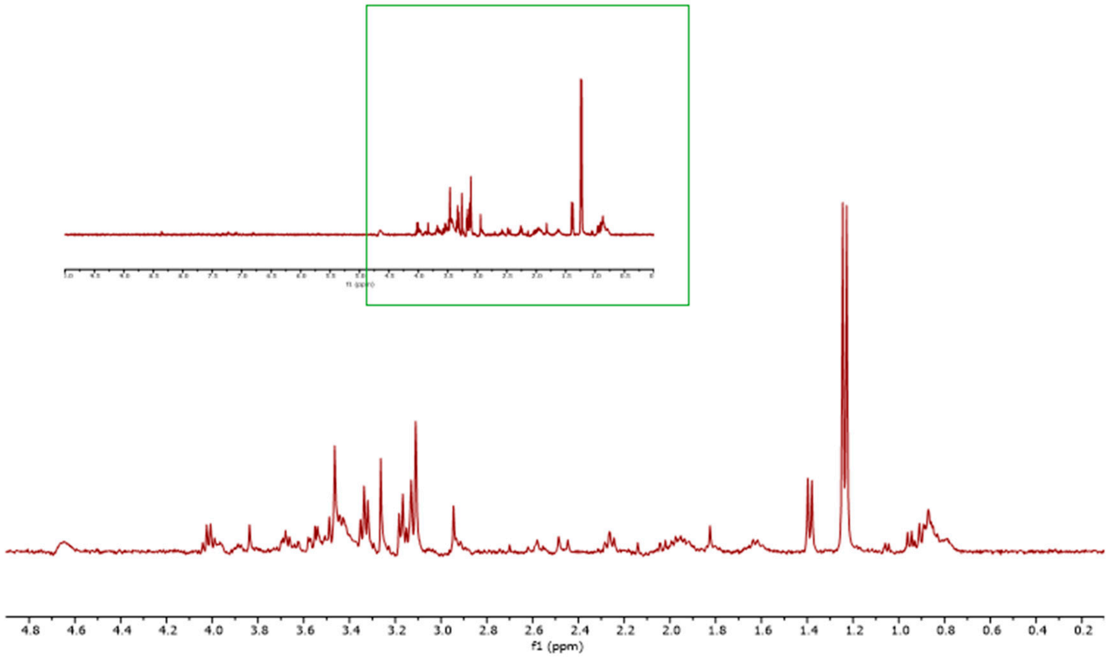

Figure S7. Representative ^{1}H NMR spectra of water/methanol extract of LLC tumor in mouse number 7.
